# Moderate External Rotation of Tibial Component Generates More Natural Kinematics Than Internal Rotation After Total Knee Arthroplasty

**DOI:** 10.3389/fbioe.2022.910311

**Published:** 2022-07-13

**Authors:** Chaohua Fang, Yichao Luan, Zhiwei Wang, Long Shao, Tiebing Qu, Cheng-Kung Cheng

**Affiliations:** ^1^ Department of Joint Surgery, Ningbo No. 6 Hospital, Ningbo, China; ^2^ School of Biomedical Engineering, Shanghai Jiao Tong University, Shanghai, China; ^3^ Engineering Research Center of Digital Medicine, Ministry of Education, Shanghai, China; ^4^ School of Biological Science and Medical Engineering, Beihang University, Beijing, China; ^5^ Department of Orthopaedics, Beijing Chaoyang Hospital, Beijing, China; ^6^ Department of Orthopaedics, Beijing Boai Hospital, Beijing, China; ^7^ The Center of Diagnosis and Treatment for Joint Disease, China Rehabilitation Research Center, Beijing, China

**Keywords:** computational simulation, total knee arthroplasty, tibial component, rotational alignment, kinematics

## Abstract

This study aimed to investigate the influence of tibial malrotation on knee kinematics after total knee arthroplasty (TKA). A symmetric fixed-bearing posterior-stabilized prosthesis was implanted in the validated knee model with different rotational alignments of the tibial component (neutral, 3° external rotation, 5° external rotation, 3° internal rotation, and 5° internal rotation). Computational kinematic simulations were used to evaluate the postoperative kinematics of the knee joint including anteroposterior translation femoral condyles and axial rotation of tibial component during 0°–135° knee flexion. The results revealed that the neutral position of the tibial component was not the closest kinematics to the intact knee, the model with 5° external rotation of the tibial component showed the closest lateral condyle translation and tibial axial rotation, and moderate external rotation could improve the kinematics after TKA.

## Introduction

Total knee arthroplasty (TKA) has been the most common treatment for severe arthritis of knee joints for the past several decades with high survival rates; however, nearly 20% of patients were still not satisfied postoperatively because of knee pain or restricted function (Bourne et al., 2010). Rotational malalignment between the femoral and tibial components is one of the reasons, and the proportion of tibial component malrotation beyond 3° can reach 57% according to a research study using 3D-CT measurement (Cerquiglini et al., 2018). Malrotation of knee components influenced the mechanical behaviors of the knee joint, including the ligament tension, patella force, and contact stress on the polyethylene liner (Kuriyama et al., 2014), and caused patellofemoral maltracking, femoral–insert interface, anterior knee pain, patellar subluxation, excessive polyethylene wear, and even early failure of the tibial liner (Cerquiglini et al., 2018).

As for the rotational alignment in TKA, the transepicondylar axis (TEA) is universally accepted as the gold standard of femoral rotational alignment. This is partly because the TEA can represent the best approximation of the actual flexion–extension axis (FEA) of the knee (Churchill et al., 1998). However, it is still controversial with regard to the tibial rotational alignment. The correct tibial rotational alignment is usually regarded, as with the anterior–posterior axis of the tibial component, as being perpendicular to TEA at the full leg extension position. It is considered the neutral position or internal or external rotation (Akagi et al., 2004; Kim et al., 2017). The positions of the femur and tibia in the primary knee joint are changeable during gait cycles, and the tibia plateau aligns with slight external rotation compared to the femoral condyle in full leg extension (Duparc et al., 2014). Until now, there has been no TKA that can replicate the kinematics of the living knee, and the main abnormal kinematics includes decreased posterior femoral rollback, paradoxical anterior femoral translation, and reverse axial rotation of the tibia (Dennis et al., 1998). For the posterior-stabilized (PS) prosthesis of TKA, the cam-post mechanism was designed to avoid paradoxical anterior femoral translation (Arnout et al., 2015). However, the rotation between femoral and tibial components was limited because of this mechanism, which resulted in complications, such as impingement, wear, and even fracture of the post (Callaghan et al., 2002; Dolan et al., 2011; Diamond et al., 2018). Therefore, it is crucial for rotational alignment when PS prostheses are used in TKA. Moreover, the few studies focusing on knee kinematics after TKA with different tibial rotational alignment show inconsistent results (Harman et al., 2012; Hutter et al., 2013; Nakahara et al., 2015). No study has evaluated how the degree of tibial component malrotation affects the kinematics of the knee joint after TKA.

The current study aimed to investigate the effect of tibial component rotation on knee kinematics after TKA. We hypothesized that external rotational alignment of the tibial component results in kinematics closer to the intact knee.

## Materials and Methods

An intact kinematic knee model which was validated in a previous study was used (Wang et al., 2012; Fang et al., 2015). This model was built according to CT data from a healthy female volunteer with informed consent before scanning, and was approved by the local institutional review board (approval number: 12-S-70). The model included the proximal tibial bone, distal femoral bone, patella, cartilage, and meniscus. Three-dimensional solid models of a symmetric fixed-bearing PFC Sigma PS prosthesis (DePuy; Johnson & Johnson, Warsaw, IN) were constructed using reverse engineering, including a femoral component, tibial component, and tibial insert.

**FIGURE 1:**
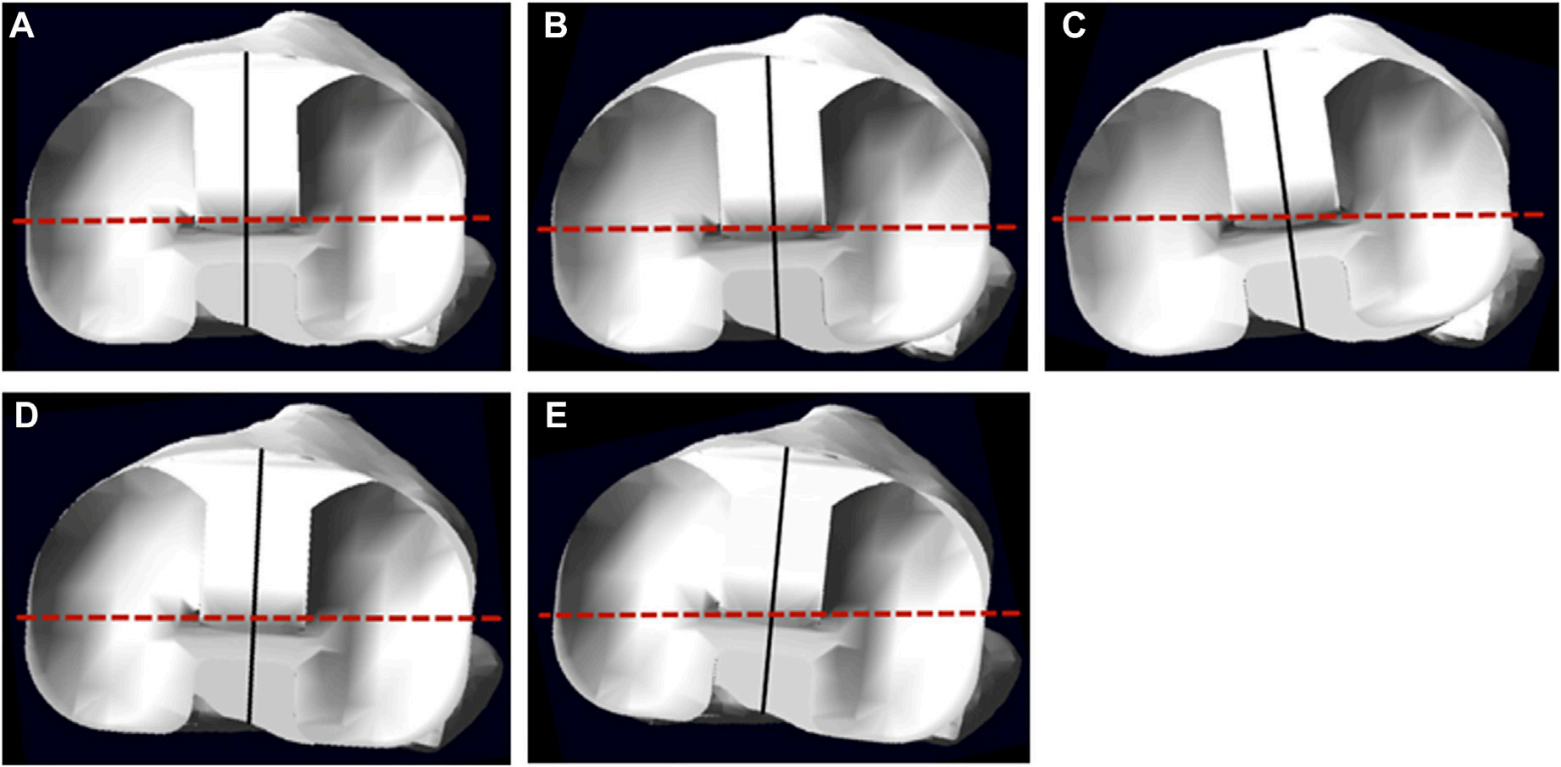
The superior view of the tibial component on the tibia; the solid line indicates the anterior–posterior axis of the tibial component, and the dotted line indicates the projection of the TEA. **(A–E)** show models A, B, C, D, and E, respectively.

The bone-cutting was performed using Pro/ENGINEER Wildfire 5.0 (Parametric Technology Corp) with the techniques of mechanical alignment and measured resection in TKA (Daines and Dennis, 2014). On the coronal and sagittal planes, the distal femoral and proximal tibia were resected perpendicular to their mechanical axes. The femoral mechanical axis was defined as the line that connected the center of the intercondylar notch and the center of the femoral head; the tibial mechanical axis was defined as the line that connected the center of the tibial plateau and the center of the talus (Wu et al., 2002). On the transverse plane, the femoral component was implanted with its transverse axis parallel to the TEA, and the tibial component was implanted depending on the medial angle of its anterior–posterior axis (the line connected the midpoints of anterior and posterior edges) and projection of the TEA on the tibial cutting surface at the full leg extension position. It was regarded as internal rotation if the angle was less than 90°, otherwise, as external rotation. Model A has an angle of 90° that was regarded as the neutral rotational alignment of the tibial component (Akagi et al., 2004; Kim et al., 2017); the other four models (Model B–E) were established with 5° internal rotation, 3° internal rotation, 3° external rotation, and 5° external rotation, respectively (Figure 1).

**FIGURE 2:**
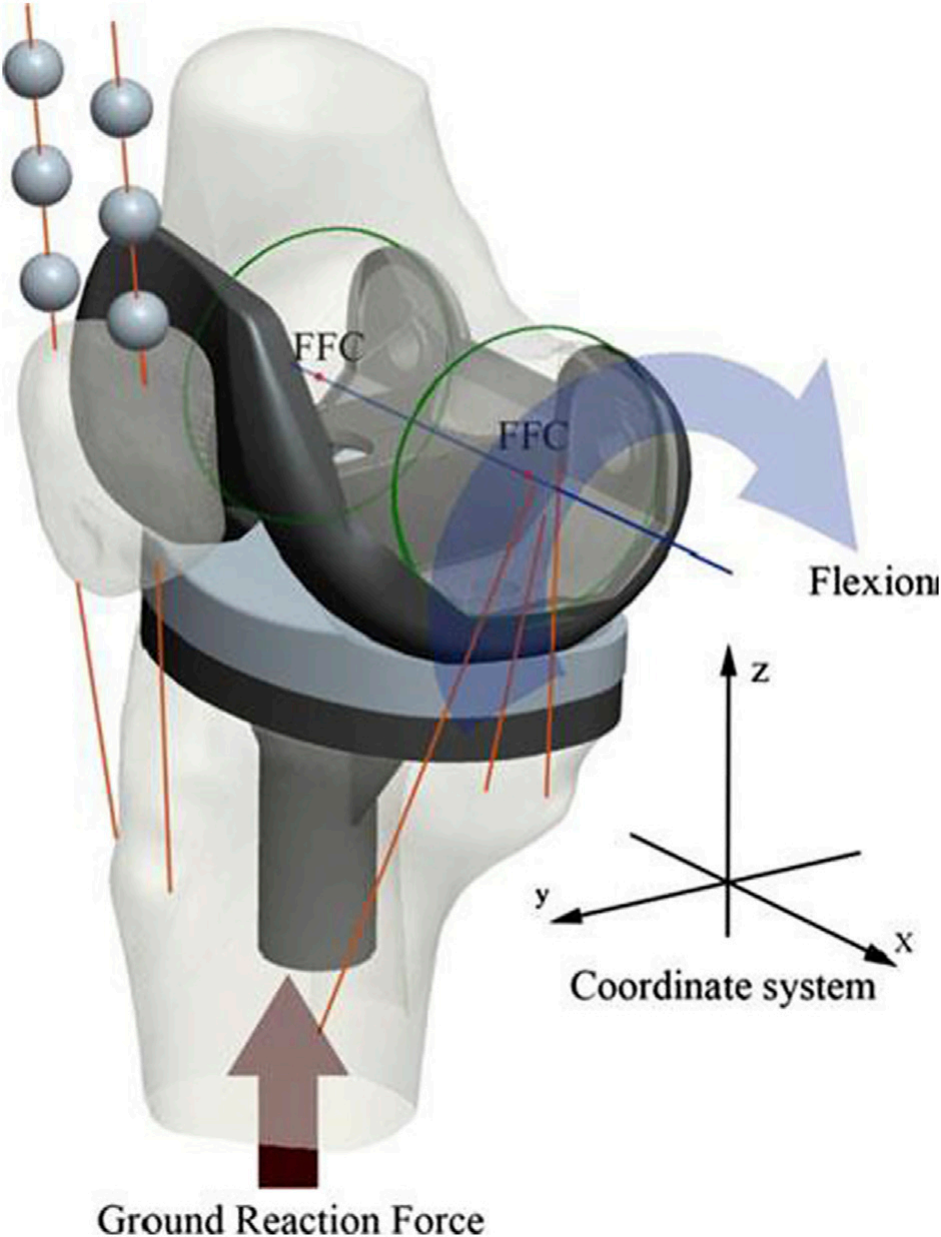
The TKA model and the coordinate system in ADAMS.

The model was assembled by placing the most distal points of the femoral condyles on the lowest points of the polyethylene tibial insert in MSC.ADAMS_R3 (MSC Software, Santa Ana, CA). The medial collateral ligament (MCL), lateral collateral ligament (LCL), cruciate ligaments, patella tendon, quadriceps, and hamstrings were simulated as nonlinear force elements to calculate knee kinematics. The origin and insertion points of ligaments and tendons were referenced from relevant literature (Ikeuchi et al., 2007; Tao et al., 2014) and confirmed by the senior surgeon (TB Qu). The flexion facet center (FFC) was generated by the circular fitting of the condyles of the femoral component (Iwaki et al., 2000). The line connecting the medial and lateral FFC was regarded as the *x*-axis, the mechanical axis was designed as the *z*-axis, and the *y*-axis was generated automatically according to the *x*-axis and *z*-axis. A Cartesian coordinate system was constructed on the original position (Grood and Suntay, 1983). During knee flexion, the displacements of medial and lateral FFCs in the *y*-direction were used to represent the anteroposterior translation of medial and lateral condyles, respectively, and tibial rotation (internal–external rotation of the tibia) was defined as the angular displacement in the *z*-direction (Figure 2).

In this model, the contact properties of tibiofemoral articulation and patellofemoral articulation were set as “solid to solid,” and the friction of these two articulations were 0.04 and 0, respectively (Godest et al., 2002). A ground reaction force (1.5 bodyweights = 750 N) was applied to the center of mass of the tibial component (D'Lima et al., 2007). The femoral component was only permitted to move in the flexion–extension direction, but the tibial component was only constrained in the flexion–extension direction and unconfined in all other directions.

All the five models were simulated with the knee flexion from 0° to 135°; the kinematics data, including femoral anteroposterior translation and tibial rotation, were acquired every 15° during knee flexion and visualized in Microsoft Excel (Version 2016; Microsoft, Redmond, WA, United States). In addition, Model A with the neutral alignment of the tibial component was used for validating the TKA model against the results of an *in vivo* kinematic study using the same prostheses and surgical techniques (Ranawat et al., 2004).

## Results

The TKA model (Model A) was validated by comparing the simulated results and *in vivo* data. The difference in both medial and lateral femoral condylar contact positions was less than 2 mm at any angle of knee flexion from 0° to 90° (Wang et al., 2012; Steinbrück et al., 2016).

The data of femoral anteroposterior translation and tibial rotation were acquired from intact knee and TKA models. Different kinematic results were generated from the intact knee model and all TKA models, especially for the lateral femoral condyle translation in the full flexion process and tibia axial rotation beyond 60° flexion.

**FIGURE 3:**
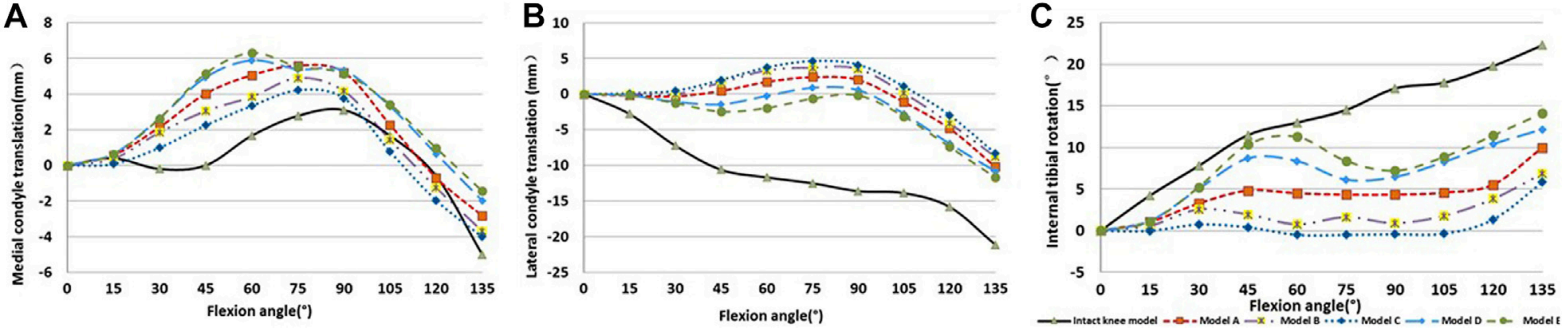
The kinematics of TKA models and intact knee model. **(A)** Medial condyle translation, **(B)** lateral condyle translation, and **(C)** internal tibial rotation. Positive means anterior translation or tibia internal rotation against the femur. Negative indicates the opposite.

The TKA models showed less posterior translation of the lateral femoral condyle and internal tibial rotation. As for the kinematics among the TKA models with different rotational alignments, the femoral condyle translation and tibial axial rotation were proportional to the external rotation of the tibial component. However, the overall trends of translation and rotation were similar. Compared with the neutral position, the internal rotation of the tibial component decreased the anterior translation of medial condyle, but increased the anterior translation of lateral femoral condyle, and decreased the internal rotation of the tibia during flexion. External malalignment showed contrary results. Moreover, the kinematic results of Model E with 5° external malrotation were the closest to the intact knee model including lateral femoral condyle translation and tibial axial rotation (Figure 3).

## Discussion

To investigate the influence of tibial rotational alignment on the kinematics of TKA, dynamic simulations of TKA models with different tibial rotational alignments were used to calculate the femoral condylar translation and tibial rotation. The key finding of this study is that the external rotational alignment of the tibial component can restore the lateral femoral condyle translation and tibial axial rotation, which was closer to a normal knee joint than the neutral position. For the medial femoral condyle translation, the internal rotational alignment is most similar to that of a normal knee. Considering the posterior translation of the lateral condyle is greater than the anterior translation of the medial condyle, the whole femoral condyle slides more posteriorly at external 5° rotational alignment than that at internal 5° rotation during flexion. However, anterior translation of the medial condyle was decreased because of internal malrotation that leads to paradoxical tibial axial rotation. Overall, the external 5° rotational alignment is better than the neutral position because it is closer to the normal joint in femoral condyle translation and tibial axial rotation. The reason for this might be that the tibia was externally rotated to the femur at full extension of the intact knee joint, and it is the tibial component implanted with a moderate external rotation that mostly aids recovery in this situation.

The goal of TKA is not only to alleviate pain and improve function but also to help the patient regain normal kinematics of the knee joint. Recovering the optimal rotational alignment of the tibial component is helpful to achieve this goal, but it still requires further research. Previous studies revealed internal malrotation can lead to many complications such as stiffness, anterior knee pain, and extensor mechanism deficiency (Bédard et al., 2011; Steinbrück et al., 2016; Abdelnasser et al., 2020). Recently, a systematic review confirmed that excessive internal rotation (>10° of internal rotation demonstrated the common value) of the tibial component was a significant risk for knee pain and inferior functional outcomes after TKA, although external rotation does not affect the results (Panni et al., 2018). External rotation might be helpful. A retrospective study of 1,696 consecutive patients (3,048 knees), with a mean follow-up duration of 15.8 years (range, 11–18 years), found that the risk factors for failure of the components involved <2° external rotational alignment of the tibial components and recommended that the tibial component be placed with the rotational alignment of 2–5° external rotation (Kim et al., 2014).

The altered postoperative kinematics might be responsible for postoperative complications and patients’ subjective dissatisfaction. The kinematics of TKA was affected by a variety of factors, such as the prosthesis design and surgery procedure (Koh et al., 2019; Wang et al., 2019). However, only a few cadaveric and computational simulation studies have investigated the influence of tibial rotational alignment. A cadaveric study recorded the kinematics of the femorotibial joint with an ultrasonic-based motion analysis system using the tibial component with 3° internal rotation, neutral, or 3° external rotation. With the regression coefficients from the mixed-effects model analysis, we found that the mean anterior translation of the femur was −0.9, 0, and 0.4 mm at the tibial component with 3° internal rotation, neutral, or 3° external rotation position, respectively, and the internal femorotibial rotation was 1.2°, 0°, and 0.2°, respectively (Steinbrück et al., 2016). These results are consistent with those of our current study, where the femoral component anterior translation increased and internal tibial axial rotation decreased if the tibial component was internally rotated. Another cadaveric study compared the preoperative and postoperative tibiofemoral kinematics from 0° to 90° of flexion with the tibial components self-adapted, 6° internal rotation, 6° external rotation and with the femoral component ligament balanced, 3° external rotation, 6° external rotation, or 6° internal rotation. The largest kinematic differences between knees were found from the combination of femoral component internal and tibial component external rotation. The tibial component with 6° external rotation can restore a tibial longitudinal rotation most similar to that of the preoperative one with the femoral component of balanced ligament, 6° external rotation. The femoral component rotation was referenced with the posterior condylar line, which is internally rotated concerning both the surgical and anatomic TEA, with mean angles between 3° and 7° in the study (Maderbacher et al., 2017). Consequently, a 6° external rotation of the femoral component might result in a neutral rotation similar to the femoral rotational alignment in the current study. It is consistent with the current study that the tibial component with 5° external rotation regains the tibial axial rotation closest to the normal knee joint. According to the literature we retrieved, only one computational simulation investigated the influence of tibial rotational alignment on tibiofemoral kinematics. For the PS implant, an externally rotated 15° tibial component permitted greater anterior femoral translation than an internally rotated 15° tibial component, and the anterior translations of the medial condyle appeared to be increased in the cases of external tibial component rotation, which is also consistent with the result in the current study (Thompson et al., 2011).

Increased MCL force resulting from the internal rotational alignment of the tibial component might be one of the possible reasons for the decreased anterior translation of the medial condyle, but MCL force also increased slightly with external rotational alignment; the orientation of the MCL might induce this discrepancy. As proven by MRI studies, the tibial attachment to the MCL is located more anteriorly than the femoral attachment in knee extension (Thompson et al., 2011). So, an internally rotated tibial component results in an internally rotated femur and posterior translational femoral medial condyle relative to the tibia, which lengthens the MCL and increases the tensile force. On the contrary, an externally rotated tibial component leads to the anterior translation of the medial femoral condyle; the length of MCL might be shortened slightly or unchanged; moreover, the MCL force would not change significantly. Meanwhile, the LCL is less affected by malrotation of the tibial component because of its lower stiffness value and is modeled as a single bundle compared to the MCL with anterior, deep, and oblique bundles. In addition, the orientation of LCL was almost straight from the beginning to the end, which was different from the MCL, so the length was less influenced by malrotation of the tibial component (Kuriyama et al., 2014).

However, there are still some limitations in the current simulation. First, only five rotational alignments were simulated. The influence of tibial malalignment on kinematics might be more specific with more simulations of different alignments. The value of malrotation was set according to a previous work in which the degree of tibial malrotation was measured referencing the Akagi line, medial 1/3 tibia tubercle, or the posterolateral corner-locked (PLCL) technique in the normal Chinese population (Fang et al., 2020). According to a retrospective cohort study with 3,048 knees, 2–5° external rotation of the tibial component accounted for 2,490 knees, which was shown as the main rotational alignment in clinical practice (Kim et al., 2014). In addition, the prosthesis used in the current study is the PFC Sigma fixed-bearing prosthesis, which is a PS prosthesis with a cam-post mechanism. Excessive internal rotation (10° internal rotation) of the tibial insert resulted in impingement between the cam-post and higher stress on the post (Huang et al., 2006). Thus, moderate malrotation alignment (5°) was used in this study, but the cutoff was not determined in the current study. Another limitation is that the results of the current study may not apply to other commercial prostheses because the posterior cruciate-retaining and insert conformity design may affect the kinematics as well. Finally, the bone model was constructed according to data from an individual, which cannot reflect on all patients because of anatomical differences.

## Conclusion

The tibial component rotational alignment can alter the kinematics after TKA, and the neutral position is not the optimal tibial rotational alignment. Moderate external rotation (5°) can restore the lateral femoral condyle translation and tibial axial rotation to a state closest to the normal knee joint compared with external 3°, internal 3°, internal 5° rotation, and neutral position. More studies that focus on the optimal rotational alignment of the tibial component, rather than the accuracy of rotational alignment, need to be conducted in the future.

## Data Availability

The raw data supporting the conclusions of this article will be made available by the authors, without undue reservation.

## Abbreviations

FEA, flexion–extension axis; FFC, flexion facet center; LCL, lateral collateral ligament; MCL, medial collateral ligament; PS, posterior-stabilized; PLCL, posterolateral corner-locked; TEA, transepicondylar axis; TKA, total knee arthroplasty.
